# Cytokine expression patterns predict suppression of vulnerable neural circuits in a mouse model of Alzheimer’s disease

**DOI:** 10.1101/2024.03.17.585383

**Published:** 2026-03-02

**Authors:** Dennis Chun Yin Chan, ChaeMin Kim, Rachel Y. Kang, Madison K. Kuhn, Samuel Cramer, Hayreddin Ünsal, Lynne M. Beidler, Kevin L. Turner, Thomas Neuberger, Nanyin Zhang, Elizabeth A. Proctor

## Abstract

Alzheimer’s disease is a neurodegenerative disorder characterized by progressive amyloid plaque accumulation, tau tangle formation, neuroimmune dysregulation, synapse and neuron loss, and changes in neural circuit activation that lead to cognitive decline and dementia. Early molecular and cellular disease-instigating events occur 20 or more years prior to presentation of symptoms, making them difficult to study, and for many years amyloid-β, the aggregating peptide seeding amyloid plaques, was thought to be the toxic factor responsible for cognitive deficit. However, strategies targeting amyloid-β aggregation and deposition have largely failed to produce safe and effective therapies, and amyloid plaque levels poorly correlate with cognitive outcomes. However, a role still exists for amyloid-β in the variation in an individual’s immune response to early, soluble forms of aggregates, and the downstream consequences of this immune response for aberrant cellular behaviors and creation of a detrimental tissue environment that harms neuron health and causes changes in neural circuit activation. Here, we perform functional magnetic resonance imaging of awake, unanesthetized Alzheimer’s disease mice to map changes in functional connectivity over the course of disease progression, in comparison to wild-type littermates. In these same individual animals, we spatiotemporally profile the immune milieu by measuring cytokines, chemokines, and growth factors across various brain regions and over the course of disease progression from pre-pathology through established cognitive deficit. We identify specific signatures of immune activation predicting hyperactivity followed by suppression of intra- and then inter-regional functional connectivity in multiple disease-relevant brain regions, following the pattern of spread of amyloid pathology.

## Full Text Availability

The license terms selected by the author(s) for this preprint version do not permit archiving in PMC. The full text is available from the preprint server.

